# Evidence of Lineage 1 and 3 West Nile Virus in Person with Neuroinvasive Disease, Nebraska, USA, 2023

**DOI:** 10.3201/eid3010.240595

**Published:** 2024-10

**Authors:** Emily Davis, Jason Velez, Jeff Hamik, Kelly Fitzpatrick, Jacki Haley, Jeremy Eschliman, Amanda Panella, J. Erin Staples, Amy Lambert, Matthew Donahue, Aaron C. Brault, Holly R. Hughes

**Affiliations:** Centers for Disease Control and Prevention, Fort Collins, Colorado, USA (E. Davis, J. Velez, K. Fitzpatrick, A. Panella, J.E. Staples, A. Lambert, A.C. Brault, H.R. Hughes);; Nebraska Department of Health and Human Services, Lincoln, Nebraska, USA (J. Hamik, M. Donahue);; Two Rivers Public Health Department, Kearney, Nebraska, USA (J. Haley, J. Eschliman)

**Keywords:** West Nile virus, viruses, zoonoses, lineage 3, Rabensburg virus, co-infection, West Nile neuroinvasive disease, genomic surveillance, vector-borne infections, United States

## Abstract

West Nile virus (WNV) is the most common cause of human arboviral disease in the contiguous United States, where only lineage 1 (L1) WNV had been found. In 2023, an immunocompetent patient was hospitalized in Nebraska with West Nile neuroinvasive disease and multisystem organ failure. Testing at the Centers for Disease Control and Prevention indicated an unusually high viral load and acute antibody response. Upon sequencing of serum and cerebrospinal fluid, we detected lineage 3 (L3) and L1 WNV genomes. L3 WNV had previously only been found in Central Europe in mosquitoes. The identification of L3 WNV in the United States and the observed clinical and laboratory features raise questions about the potential effect of L3 WNV on the transmission dynamics and pathogenicity of WNV infections. Determining the distribution and prevalence of L3 WNV in the United States and any public health and clinical implications is critical.

West Nile virus (WNV) is a flavivirus within the family Flaviviridae. Since WNV was identified in New York, USA, in 1999, it has become the leading cause of arboviral disease in the contiguous United States (*1*–*3*). WNV is maintained in a transmission cycle between mosquitoes and birds, in which infection can range from asymptomatic to lethal depending on the avian species (*4*). Similarly, in dead-end hosts, such as humans, disease severity varies. Most human WNV infections are asymptomatic; however, <1% of infections result in severe neurologic disease (*3*). WNV disease risk generally increases with age and underlying conditions (*5*).

The diagnosis of WNV disease is typically made on the basis of clinical symptoms and serologic testing because viremia is typically transient and low titer. WNV IgM is detected by using immunosorbent assays, and diagnosis is confirmed with a plaque reduction neutralization test. In persons who are immunosuppressed, or when serologic findings are not conclusive, molecular detection of WNV RNA in serum or cerebrospinal fluid (CSF) can be used to make the diagnosis (*6*)

Up to 9 distinct lineages of WNV have been proposed on the basis of genotypic analyses of the envelope and nonstructural protein 5 genes (*1*,*7*–*11*). Sublineage 1a is broadly distributed in Africa, Europe, and the Americas. Lineage 2 (L2) WNV was primarily found in sub-Saharan Africa until the early 2000s, when it rapidly emerged in Europe. Many WNV lineages are referred to by other names, including Kunjin (L1b) (*12*), Koutango (L7) (*13*), and Rabensburg (L3) viruses (*14*,*15*).

Murine virulence studies and clinical testing of humans has shown that L1 and L2 can cause neuroinvasive disease (*16*,*17*). In contrast, L3 WNV has not been found to cause disease or pathology in birds or mammals, being detected only in mosquito pools in the Czech Republic (*7*,*15*,*18*,*19*). The restricted host range of L3 WNV was confirmed in experiments in which viremia and antibodies were not detected after avian infection (*14*). Furthermore, the virus did not replicate in mammalian cell culture at physiologic temperatures and was highly attenuated in adult mouse models (*14*,*18*–*20*).

In 2023, an immunocompetent patient was hospitalized in Nebraska, USA, with West Nile neuroinvasive disease and multisystem organ failure. Testing at the Centers for Disease Control and Prevention (CDC) indicated an unusually high viral load. The high viremia prompted genomic surveillance testing to investigate whether mutations in L1 WNV could potentially explain the high viremia findings. High-throughput sequencing (HTS) indicated the presence of L1 and L3 WNV RNA in the patient’s serum and CSF. In this article, we describe the clinical features and course of disease in the patient and the initial virologic findings that might affect the transmission dynamics and pathogenicity of WNV infections.

## Materials and Methods

### Case Information

We collected case information as part of surveillance and follow-up of a nationally notifiable disease. We conducted interviews to determine potential travel and exposure history and obtained clinical information from the patient and healthcare providers.

### Case-Patient Clinical Description

A man 70–79 years of age who had coronary artery disease, hyperlipemia, controlled type 2 diabetes mellitus, obesity, and mild chronic kidney disease was in his usual state of health until mid-August 2023, when he had onset of fever, myalgias, diarrhea, headache, dyspnea on exertion, and decreased appetite. Four days after symptom onset, the patient visited a local hospital, where he was noted to have increased inflammatory markers (C-reactive protein 17 mg/dL [reference range <0.3 mg/dL], erythrocyte sedimentation rate 43 mm/h [reference range 0–15 mm/h], and procalcitonin 1.48 ng/mL [reference range <0.1 ng/mL]), as well as leukopenia (leukocytes 2,700 cells/μL [reference range 4,000–11,000 cells/μL]) and thrombocytopenia (platelets 115,000/μL [reference range 150,000–450,000/μL]). He was hospitalized and given ceftriaxone. 

Two days after admission, he continued to have fevers with increasing headaches and neurologic signs and symptoms, including bilateral fine tremors in his hands, decreased strength, slower gait, stiff neck, and difficulty in responding to questions. A lumber puncture revealed an decreased leukocyte count (1,939 cells/mm^3^ [reference range 4,000–11,000 cells/mm^3^]) with a neutrophilic predominance (72%), elevated protein (242 mg/dL [reference range 60–83 mg/dL], and <3,000 erythrocytes cells/mm^3^ (reference range 3.93–5.96 million erythrocytes /mm^3^); glucose was within reference range (54 mg/dL [reference range 50–75 mg/dL]). The patient was transferred to the intensive care unit, and his antimicrobial drug treatment regimen was broadened to include vancomycin, meropenem, acyclovir, and doxycycline.

The patient became more confused and then unresponsive and had onset of ascending paralysis to his thoracic region; seizure-like activity was noted on day 3 of hospitalization. A contrast magnetic resonance imaging of his spine and brain had no acute findings, and an electroencephalogram revealed nonlocalized cerebral dysfunction without seizures. He was transferred to a tertiary-care center the following day (day 8 after illness onset), where he was intubated and found to have acute kidney injury. The patient remained critically ill on a ventilator until his mental status began to improve on hospital day 9. He eventually had a percutaneous endoscopic gastrostomy tube placed, and a tracheostomy was performed before the patient was transferred to a long-term care hospital and then a skilled nursing facility, where he remained for >3 months.

Two weeks before illness onset, the patient had traveled to northeast Colorado for 2 nights, but otherwise he did not have other domestic or international travel. He reported no known mosquito or tick bites when recreating outdoors, which he did often. He did not have pets or exposure to other animals.

### Serologic Testing

We had ELISA testing performed at a commercial reference laboratory. The laboratory then sent positive serum and CSF samples to the CDC Arboviral Diseases Branch (Division of Vector-Borne Diseases, National Center for Emerging and Zoonotic Infectious Diseases; Fort Collins, CO, USA) for confirmation, where we performed plaque-reduction neutralization tests as previously described (*21*). In brief, we diluted an aliquot of the patient’s serum sample 1:5 before 2-fold serial dilutions, whereas the CSF starting dilution was 1:2. We incubated these dilutions with 100 PFUs of L1 WNV (strain NY99) and used them to infect Vero cells followed by an agarose overlay. After 3 days, we placed an overlay including neutral red on top of the monolayer and counted plaques the next day. The diagnostic cutoff for positivity was a 90% reduction in PFUs.

### Molecular Testing

We extracted viral RNA from clinical samples by using the QIAmp Viral RNA Mini Kit (QIAGEN, https://www.qiagen.com). We followed the manufacturer’s protocol unless otherwise stated. We determined input and elution volume on the basis of sample availability (for serum, input 500 µL and elution 60 µL; for CSF, input 80 µL and elution 50 µL). We completed an additional low-volume extraction on the remaining volume of serum (20 µL input and 30 µL elution volume). To confirm a laboratory contamination event had not occurred, we performed the CSF and second serum extraction in a separate laboratory that only handled bacteria and where WNV had never been present. We performed real-time reverse transcription PCR (rRT-PCR) by using the QuantiTect Probe RT-PCR Kit (QIAGEN) and L1-specific (*1*) and L3-specific (*22*) primers according to the manufacturers’ protocols.

### Virus Isolation

We grew and maintained Vero cells at 37°C as previously described (*23*). We inoculated cell monolayers with 200 µL of the patient’s serum and monitored them daily for cytopathic effects (CPE). At 3 days after inoculation, 50% of the cells demonstrated CPE and we harvested an isolate (Vero passage 1 [Vp1]). We centrifuged cell supernatant to clear cell debris, then aliquoted and stored it at −80°C. We extracted RNA by using an input of 100 µL and used an elution of 100 µL for rRT-PCR testing, as described.

We inoculated Vp1 onto Vero cells at 32°C and 28°C, allowed it to incubate for 1 hour, and then overlaid it. After 3 days, we added to a second overlay to the wells, including neutral red. We monitored plates for plaque formation for 11 days. We picked plaques and suspended them in BA-1 diluent before using this inoculum to inoculate Vero cells, and we monitored CPE as described previously. We also isolated RNA as described.

### Next-Generation Sequencing and Analysis

We generated complementary DNA (cDNA) by using the Ovation RNA-Seq System V2 (Tecan Life Sciences, https://lifesciences.tecan.com). We prepared sequencing libraries by using the Nextera XT DNA Library Prep Kit (Illumina, https://www.illumina.com) and IDT DNA/RNA UD indexes (IDT, https://www.idtdna.com). We completed sequencing on RNA extracted from the clinical samples and Vp1 by using the NextSeq1000 and a P1, 300-cycle kit or a MiSeq and a V2 300-cycle kit (Illumina).

We completed de novo assembly by using SPAdes version 3.15.3 (https://github.com/ablab/spades) and its RNA viral presets. We searched the resulting contigs for viral origin by using the viral_nt database and CLI of BLASTn version 2.12.0 (https://ftp.ncbi.nlm.nih.gov/blast/executables/LATEST) and confirmed them by using the nucleotide BLAST database (https://blast.ncbi.nlm.nih.gov). To resolve areas of overlap between WNVs lineages, we also completed reference alignment and used it to generate consensus sequences as previously described (*24*). We used Bowtie2 version 2.2.5 (https://github.com/BenLangmead/bowtie2) to align samples to references by using very-sensitive-local presets. We used Samtools version 1.15.1 (https://github.com/samtools/samtools/releases) to sort reads by coordinate, from which we then removed duplicates by using Picard version 2.23.0 https://github.com/broadinstitute/picard/releases). We calculated coverage by using Bamtools version 2.5.2 (https://github.com/pezmaster31/bamtools).

## Results

### Case-Patient Testing

For the case-patient, testing for various bacterial and viral pathogens was negative on the serum and CSF samples (Appendix Table 1). Samples of serum collected 4 days and CSF collected 6 days after illness onset were tested by using WNV IgM ELISA at a commercial reference laboratory. Both serum and CSF samples were identified to be IgM-positive.

### Diagnostic Evaluation of Presumptive WNV Infection

To confirm the WNV IgM results, serum and CSF samples were sent to CDC’s Arboviral Diseases Branch, where we conducted plaque reduction neutralization testing. We observed reduced plaque sizes compared with control WNV L1 plaques, and the degree of neutralization did not meet the cutoff for positivity for either serum or CSF. However, we confirmed WNV infection by using the L1-specific WNV rRT-PCR assay with an average cycle threshold (Ct) of 21.3 on serum. By using an on-plate standard curve of L1 WNV RNA (R^2^ = 0.9802), we calculated that this Ct approximated 5.5 log_10_ PFU equivalents of L1 WNV (Appendix Figure). We detected L1 WNV RNA again in Vp1 by using rRT-PCR (Ct 11, estimated titer 8.9 log_10_ PFU equivalents). Volume did not allow for L3-specific molecular detection to be performed on the serum sample. Results of a retrospective rRT-PCR test using L3 primers on the Vp1 sample was negative.

### Metagenomic Sequencing Confirmation of L1 and Detection of L3 WNV RNA

**Figure 1:**
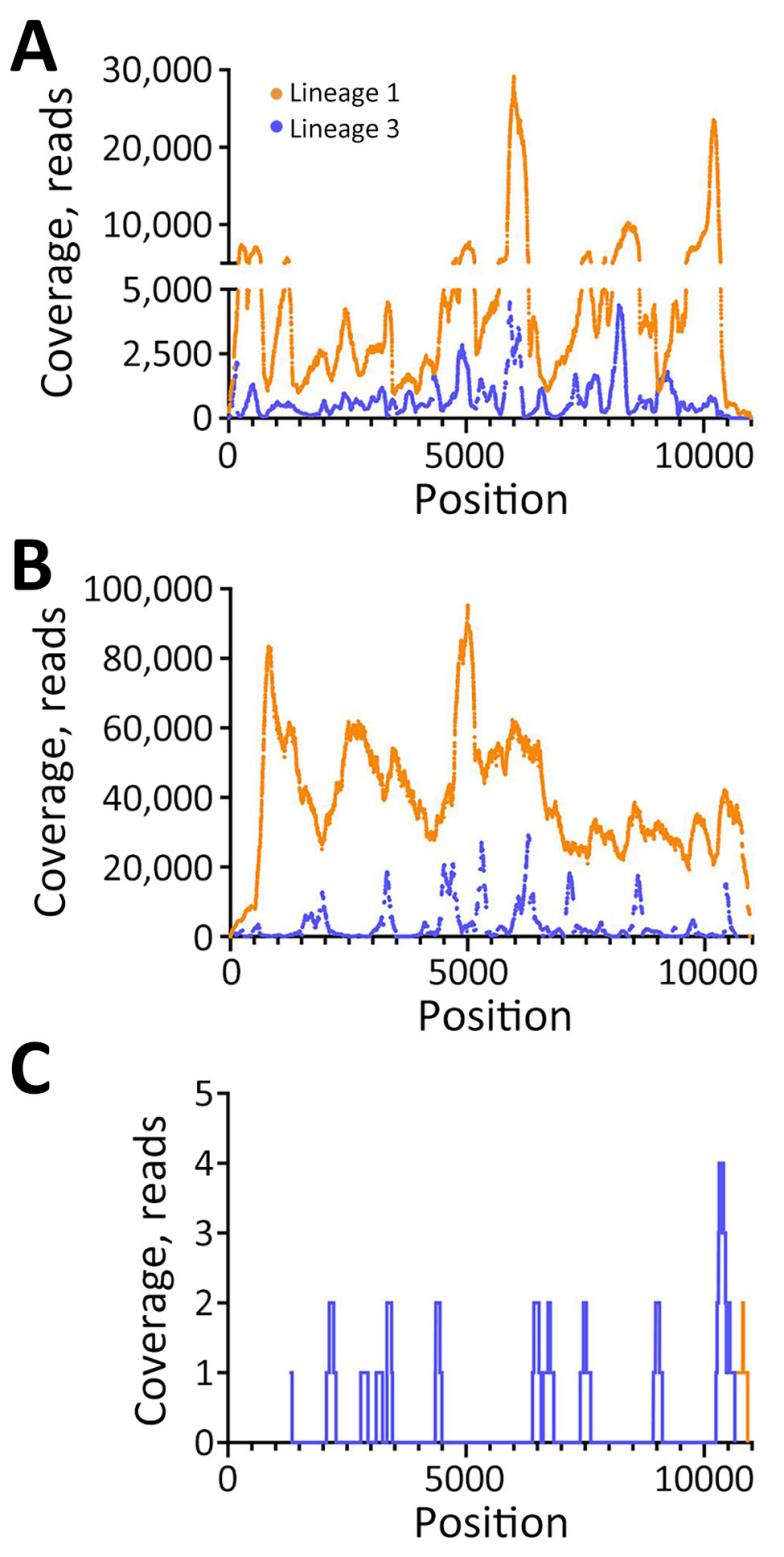
Coverage of lineage 1 and lineage 3 WNV as determined by reference guided assembly in samples from a patient with neuroinvasive disease and evidence of lineage 1 and 3 WNV infection, Nebraska, USA, 2023. Using de novo assembled consensus sequences, a reference guided assembly was completed. Reads mapped to lineage 1 (orange) and lineage 3 (purple) WNV are shown in serum (A), after Vero passage 1 (B), and in cerebrospinal fluid (C). WNV, West Nile virus.

We used RNA from serum and Vp1 to perform metagenomic sequencing on the NextSeq1000 platform. In serum, we detected full-length L1 WNV (4,299,218 total reads, 4,505 average reads/base; GenBank accession no. PP445211) and full-length L3 WNV (542,849 reads, 1,012 average reads/base; GenBank accession no. PP445212) by de novo assembly (Figure 1, panel A). We completed a second, low-volume extraction to confirm the presence of L3 WNV RNA in the serum that had undergone freeze-thaw and sequenced it on the MiSeq platform. We detected L1 and L3 WNV RNA by using de novo assembly (2,038–nt long contig of L1 WNV and 510-nt contig of L3) and subsequent reference guided assembly (1,376 reads for L1 and 334 reads for L3). We detected full-length L1 WNV RNA by using de novo assembly in Vp1 (27,347,544 total reads and 39,600 average reads/base) (Figure 1, panel B). We compared both serum and Vp1 sequences of L1 with NY99 (GenBank accession no. MZ605381); the serum sequence had 98.7% nucleotide identity and the Vp1 sequence had 98.6% nucleotide identity. We detected L3 WNV in Vp1 (97.3% genome coverage) through reference guided assembly (380,953 reads and 3,326 average reads/base) (Figure 1, panel B; Appendix Table 4). We sequenced RNA from the CSF on the MiSeq platform. We detected only 2 reads of L1 WNV RNA (Figure 1, panel C), mapping to 10,679–10,909 nt. We detected 20 reads of L3 WNV (18.8% genome coverage) (Figure 1, panel C).

**Figure 2:**
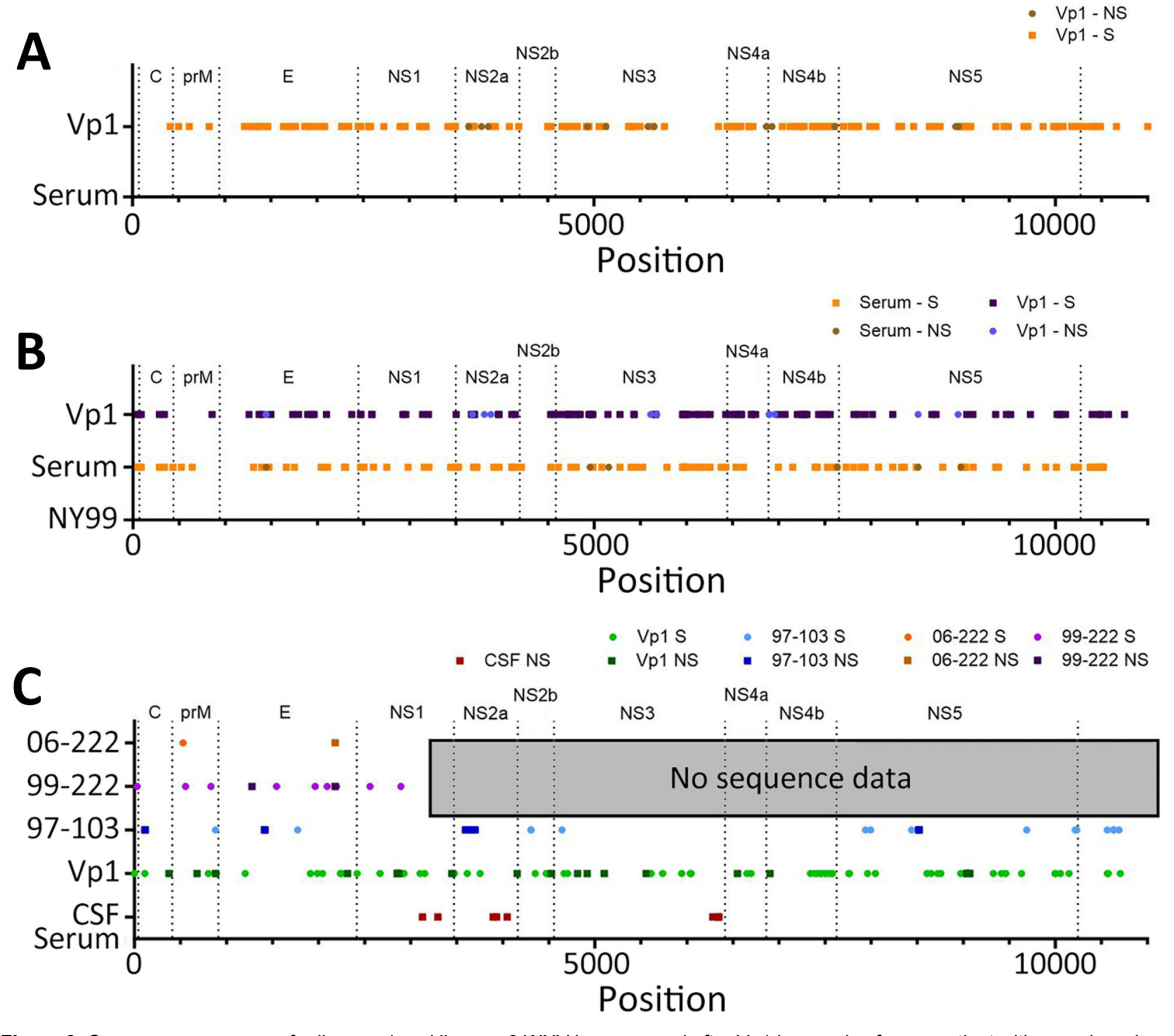
Consensus sequences for lineage 1 and lineage 3 WNV in serum and after Vp1 in samples from a patient with neuroinvasive disease and evidence of lineage 1 and 3 WNV infection, Nebraska, USA, 2023. Lineage 1 and lineage 3 WNV are distinct at the consensus level compared with Vp1 and historical strains. A, B) The consensus sequence of lineage 1 WNV in serum was compared with that of Vp1 (A), and those 2 sequences were then compared with the prototypical North American lineage 1 WNV strain, NY99 (B). C) Serum lineage 3 WNV consensus sequence was compared with the partial lineage 3 WNV sequences determined from Vp1 and CSF and historical lineage 3 WNV strains. C, capsid; CSF, cerebrospinal fluid; E, envelope; NS, nonstructural; prM, premembrane; Vp1, Vero passage 1; WNV, West Nile virus.

Comparison of the serum and Vp1 L1 WNV revealed 134 synonymous nucleotide changes, 12 nonsynonymous nucleotide changes, and 7 changes within the 3′ untranslated region (UTR), which corresponds to 98.6% nucleotide identity (Figure 2, panel A; Appendix Table 2). Comparison of the serum L3 WNV RNA and the L3 WNV detected in Vp1 revealed 1 change in the 5′ UTR, 68 synonymous nucleotide changes, 21 nonsynonymous nucleotide changes, and three 3′ UTR changes, which equates to 97.2% nucleotide identity between L3 detected in serum and Vp1 (Figure 2, panel B; Appendix Table 3).

We compared the consensus sequence of the serum L3 sequence with historical strains of L3 WNV: 97-103 (GenBank accession no. AY765264, isolated in 1997), 99-222 (GenBank accession no. GQ421359, isolated in 1999), and 06-222 (GenBank accession no. GQ421358, isolated in 2006). Compared with 97-103, the only available full-length sequence of L3 in GenBank, the serum L3 WNV strain was 99.1% identical at the nucleotide level (8 synonymous changes, 5 nonsynonymous changes, 1 change in the 5′ UTR, and 2 changes in the 3′ UTR). The serum L3 WNV strain was 99.6% identical at the nucleotide level with the partial sequences of 99-222 and 99.9% identical at the nucleotide level with the partial sequences of 06-222 (Figure 2, panel C; Appendix Table 5).

When the partial consensus sequence of L3 detected in CSF was compared with L3 WNV detected in the serum, we detected 9 nucleotide changes, all resulting in amino acid substitutions (Figure 1, panel C; Appendix Table 6). The contig mapping to L1 had 100% identity to that of the serum L1 WNV.

### Plaque Pick Isolation of L1 and L3 WNV at Low Temperature

We selected 3 plaques for HTS on the basis of the time needed for visualization after neutral red overlay. All plaque picks (Pp) were positive for L1 by rRT-PCR. None were positive by L3 specific rRT-PCR. Pp1 was similar to the expected WNV plaque phenotype but appeared at 3 days after overlay (1 day later than normal) and was picked from plates incubated at 32°C. Pp2 also was derived from a plate incubated at 32°C but appeared 9 days after overlay. Pp3 was picked from cells held at 28°C and appeared 7 days after overlay. Upon reference guided assembly, we detected L3 WNV in all plaques at a much lower rate than L1 WNV (Appendix Table 7). 

## Discussion

We describe evidence of L3 WNV in the United States and L3 WNV detection in an immunocompetent patient’s samples with atypical diagnostic test findings. The patient had detectable WNV IgM antibodies without sufficient neutralizing antibodies to be considered WNV positive on a day 4 serum and day 6 CSF samples. We did not collect convalescent samples to test for a delayed neutralizing or a cross-reactive neutralizing response; however, L1 rRT-PCR results demonstrated an uncharacteristically low Ct value, indicating a high level of virus in the patient’s serum. HTS conducted to investigate whether mutations in L1 WNV could potentially explain the diagnostic findings indicated the presence of L1 and L3 WNV in the patient’s serum and CSF.

Viremia is transient in patients with WNV disease, and the period of viremia typically ends with development of IgM, often before symptom onset (*25*). The estimated 5.5 log_10_ PFU/mL of virus in the serum of a patient who was not taking immunosuppressive medications or known to have a medical condition that caused substantial immunosuppression is abnormal. Data from asymptomatic blood donors who screen positive for WNV RNA typically demonstrate a level of viremia <80 PFU/mL, although blood donors often can have extended RNA positivity in whole blood (*26*,*27*). It is unclear whether the ability of L1 WNV to replicate to high titer without eliciting a neutralizing antibody response is caused by an interaction with L3 virus, alterations in the immune response (given the 2 viral infections), an unknown host factor, or the timing of sample collection. However, the amount of virus present in the patient exceeds a level where mosquitoes are known to become infected in laboratory setting (*28*,*29*). Additional work will be necessary to determine if this level of virus in human blood can lead to humans playing a role in the WNV transmission cycle.

Across multiple lineages, WNV has been demonstrated to infect 75 mosquito species and 300 bird, reptile, and mammal species (*30*–*32*). This broad host range is attributed to the ability of WNV to replicate efficiently because of rapid evolution in the new host (*33*). However, L3 WNV was previously thought to exist only in the mosquito vector (*Culex pipiens* and *Aedes rossicus*), with maintenance being completely reliant on vertical transmission (*14*,*20*,*22*). Although L3 WNV was demonstrated to grow in avian cell culture (*22*), no viremia or antibodies have been detected in vivo in experiments using chickens and house sparrows (*14*). L3 WNV is unable to grow in mammalian cell culture (e.g., Vero, Vero E6, human embryonic kidney 293, and baby hamster kidney cells) at 37°C unless RNA is electroporated into cells (*22*). Furthermore, L3 WNV has been demonstrated to display restricted virulence compared with other lineages of WNV(*18*,*19*). L3 WNV causes no disease in adult outbred mice, regardless of the route of infection (including intracranial), and caused reduced disease in the highly susceptible suckling mouse model (*18*,*19*). The identification of L3 RNA in a human might have occurred through replication complex interactions between L1 and L3 WNV in co-infected cells; however, we did not test this hypothesis in our study.

Flavivirus co-infection of mosquitoes, birds, and humans has been observed in many flavivirus-endemic regions (*34*–*40*). Alterations in pathogenesis caused by dual infections is complex because groups have demonstrated both increased (*41*,*42*) and decreased (*36*,*43*) disease in cases in which 2 flaviviruses infect a host simultaneously. A study in which mosquitoes were coinfected with dengue and Zika viruses demonstrated that flaviviruses can interact through their replication complexes, substantially enhancing viral replication in the vector and vector competence (*44*). The potential for viral interaction in the patient described here is supported by identification of both L1 and L3 WNV RNA in plaque picks of Vp1 grown at low temperatures, suggesting that the co-infection of L1 and L3 result in hybrid replication complexes and the viruses are co-packaged to some degree. Supporting this theory, the partial L3 genomes detected in plaque picks corresponded to regions of high coverage observed in Vp1 L3, indicating that degraded L3 RNA was replicated and packaged with L1. The large disparity between the number of reads associated with both lineages upon sequencing does suggest that far less L3 than L1 WNV RNA was present in the clinical sample, which probably led to a failure to isolate L3 virus or detect L3 RNA by rRT-PCR in Vp1.

The L3 WNV we detected is similar to the only other complete isolate (97-103), differing at only 15 nt; of those differences, 5 were nonsynonymous. Because of nucleotide similarities between the L3 and historical strains, because the 97-103 isolate exists at CDC, and because L3 was only detected by using a very sensitive HTS (*44*), RNA was re-extracted from the clinical samples in a laboratory only conducting bacterial assays to exclude contamination issues. Although contamination of the samples before arriving at CDC cannot be excluded, the 1 other laboratory in the United States that handles L3 is in New York, a different location from where the patient samples were handled and tested. Another factor potentially supporting the finding of L3 in the clinical samples was that the L3 WNV genome detected in serum was most similar to the partial sequence from L3 WNV strain 06-222, which is not present at CDC.

The 06-222 isolate of L3 WNV was collected in 2006 and was demonstrated to be more virulent than the prototype strain of L3 WNV, 97-103, in suckling mice (*14*). Overall, it is unclear why the virus has changed so little over 26 years; however, viral evolution is necessitated by rapid replication at elevated temperatures and host-specific pressures (*22*,*45*–*49*). Because L3 WNV grows slower at lower temperatures and purportedly in fewer hosts, virus evolution might be slower. The L3 WNV we observed is more distinct from historical strains than historical strains are from themselves, which indicates some evolution has occurred, just at a slower rate than the more rapidly replicating L1 WNV. The similarity could also indicate a more recent introduction of L3 into the United States, but more work is necessary, including field work to identify where L3 virus might be circulating, to determine how the virus might have evolved and adapted to a specific ecologic niche.

The effect of dual infection with L1 and L3 WNV on the patient’s clinical course and outcome is unclear because the patient’s age and underlying conditions are risk factors for more severe WNV disease. The patient had encephalitis, required intensive care, and had multiple organ system failure. He survived but did have several sequelae requiring long-term assisted care. Of note, none of the amino acid changes in the L3 WNV RNA we have described have been associated with alterations in virulence in L1 WNV; however, molecular determinants of virulence probably differ between the 2 lineages.

Current methods of surveillance do not include assays that will detect L3 WNV by molecular testing (*14*) or differentiate L3 from L1 through serologic testing (*15*), so the distribution and prevalence of disease related to L3 infection in the United States is not known. More work is needed to be determine the effect that L3 has, either with or without concurrent infection with L1, on WNV transmission dynamics and pathogenicity. Retrospective and prospective vector surveillance efforts are planned to determine how pervasive L3 WNV is among native mosquito species. In addition, CDC is working with the Nebraska Department of Health and Human Services to determine if additional L1 and L3 infections or only L3 infections have occurred in patients with similar clinical or diagnostic findings.

Appendix Additional information about evidence of lineage 1 and 3 West Nile virus in person with neuroinvasive disease, Nebraska, USA, 2023.
