# Supplementary material for: Evidence of Lineage 1 and 3 West Nile Virus in Person with Neuroinvasive Disease, Nebraska, USA, 2023
Source: Emerg Infect Dis. 2024 Oct;30(10):2090–8. doi: 10.3201/eid3010.240595 (PMC11431902; doi:10.3201/eid3010.240595)
Supplement: Appendix — Additional information about evidence of lineage 1 and 3 West Nile virus in person with neuroinvasive disease, Nebraska, USA, 2023. [file 24-0595-Techapp-s1.pdf]

Article DOI: https://doi.org/10.3201/eid3010.240595

EID cannot ensure accessibility for supplementary materials supplied by authors. Readers who have difficulty accessing supplementary content should contact the authors for assistance.

Evidence of Lineage 1 and 3 West Nile Virus in Person with Neuroinvasive Disease, Nebraska, USA, 2023

Appendix

Appendix Table 1. Test results for infectious disease pathogens

Testing performed on serum or CSF from patient	Results
Biofire Meningoencephalitis Panel (RT-PCR)<br>*Escherichia coli K1, Haemophilus influenzae, Listeria monocytogenes, Neisseria meningitidis, Streptococcus agalactiae, Streptococcus pneumoniae*<br>Cytomegalovirus (CMV), Enterovirus (EV), Herpes simplex virus 1 (HSV-1), Herpes simplex virus 2 (HSV-2)<br>Human herpesvirus 6 (HHV-6), Human parechovirus (HPeV), Varicella zoster virus (VZV), *Cryptococcus (C. neoformans/C. gattii)*	Negative
West Nile virus IgM antibodies by ELISA	Positive for Day 6 CSF; Positive/Equivocal* for Day 4 serum
Tickborne disease PCR panel<br>*Anaplasma phagocytophilum, Ehrlichia challeensis*<br>*Ehrlichia ewingii, Ehrlichia muris eauclairensis, Babesia spp.*	Negative
Serologic testing for tickborne disease<br>*Anaplasma phagocytophilum, Ehrlichia challeensis, Rickettsia ricketsii, Babesia spp., Coxiella brunetti*	Negative
Gram stain and bacterial culture	Negative for multiple sera and CSF samples

*Sample tested positive at a commercial laboratory and equivocal for WNV IgM antibodies at the NE state laboratory.

Appendix Table 2. Nucleotide (nt) changes between L1 WNV detected in serum and L1 WNV detected in Vp1

Position	Ln1 serum nt	Vp1 nt	Position in codon	Protein	Position in protein	Ln1 serum aa	Vp1 aa
409	T	C	3	capsid	113	V	V
499	T	A	3	prM	20	T	T
616	C	T	3	prM	59	G	G
832	C	T	3	prM	131	N	N
1213	G	A	3	E	91	V	V
1282	T	C	3	E	114	D	D
1342	T	C	3	E	134	N	N
1369	T	C	3	E	143	V	V
1375	A	G	3	E	145	G	G
1447	T	C	3	E	169	I	I
1468	C	T	3	E	176	Y	Y
1636	A	G	3	E	232	V	V
1705	G	A	3	E	255	L	L
1724	T	C	1	E	262	L	L
1768	C	T	3	E	276	S	S
1867	C	T	3	E	309	F	F
1913	T	C	1	E	325	L	L
1939	T	C	3	E	333	D	D
2014	T	C	3	E	358	V	V
2071	A	C	3	E	377	P	P
2083	T	C	3	E	381	D	D
2266	T	C	3	E	442	V	V

Position	Ln1 serum nt	Vp1 nt	Position in codon	Protein	Position in protein	Ln1 serum aa	Vp1 aa
2278	T	C	3	E	446	F	F
2345	C	T	1	E	469	L	L
2446	C	T	3	ns1	1	D	D
2476	G	A	3	ns1	11	Q	Q
2560	A	G	3	ns1	39	P	P
2567	C	T	1	ns1	42	L	L
2581	C	T	3	ns1	46	I	I
2725	A	G	3	ns1	94	E	E
2900	T	C	1	ns1	153	L	L
2932	C	T	3	ns1	163	T	T
2956	T	C	3	ns1	171	V	V
3112	T	C	3	ns1	223	C	C
3130	C	T	3	ns1	229	H	H
3178	C	T	3	ns1	245	V	V
3182	T	C	1	ns1	247	L	L
3418	C	T	3	ns1	325	T	T
3475	T	C	3	ns1	344	T	T
3478	C	T	3	ns1	345	L	L
3481	A	G	3	ns1	346	V	V
3502	C	T	3	ns2a	1	Y	Y
3643	C	T	3	ns2a	48	G	G
3654	C	T	2	ns2a	52	**T**	**I**
3667	T	C	3	ns2a	56	R	R
3673	C	T	3	ns2a	58	V	V
3677	T	C	1	ns2a	60	L	L
3703	C	T	3	ns2a	68	S	S
3782	C	T	1	ns2a	95	**L**	**F**
3854	C	T	1	ns2a	119	**H**	**Y**
3886	T	C	3	ns2a	129	I	I
3934	C	T	3	ns2a	145	A	A
4084	G	A	3	ns2a	195	A	A
4186	C	T	3	ns2a	229	R	R
4501	C	T	3	ns2b	103	V	V
4504	C	T	3	ns2b	104	C	C
4538	T	C	1	ns2b	116	L	L
4654	C	T	3	ns3	23	Y	Y
4693	A	G	3	ns3	36	A	A
4699	G	A	3	ns3	38	A	A
4723	T	C	3	ns3	46	F	F
4732	T	C	3	ns3	49	L	L
4804	G	A	3	ns3	73	K	K
4822	C	T	3	ns3	79	Y	Y
4918	G	A	3	ns3	111	T	T
4929	C	T	2	ns3	115	**A**	**V**
4936	A	G	3	ns3	117	K	K
4945	A	G	3	ns3	120	E	E
5056	T	C	3	ns3	157	P	P
5122	C	T	3	ns3	179	F	F
5131	T	G	3	ns3	182	**D**	**E**
5374	C	T	3	ns3	263	H	H
5404	T	C	3	ns3	273	P	P
5407	C	T	3	ns3	274	H	H
5431	T	C	3	ns3	282	F	F
5500	T	C	3	ns3	305	V	V
5585	T	A	1	ns3	334	**S**	**T**
5632	C	T	3	ns3	349	Y	Y
5641	C	T	3	ns3	352	I	I
5652	C	T	2	ns3	356	**T**	**I**
5764	T	G	3	ns3	393	T	T
6349	G	A	3	ns3	588	E	E
6442	T	C	3	ns3	619	R	R
6496	G	A	3	ns4a	18	G	G
6514	C	T	3	ns4a	24	L	L
6517	C	T	3	ns4a	25	D	D
6562	C	T	3	ns4a	40	H	H
6572	C	T	1	ns4a	44	L	L
6592	C	T	3	ns4a	50	A	A
6679	G	A	3	ns4a	79	K	K

Position	Ln1 serum nt	Vp1 nt	Position in codon	Protein	Position in protein	Ln1 serum aa	Vp1 aa
6686	T	C	1	ns4a	82	L	L
6724	T	C	3	ns4a	94	C	C
6869	C	T	1	ns4a	143	**L**	**F**
6875	A	G	1	ns4a	145	**S**	**G**
6930	G	T	2	ns4b	14	**S**	**I**
7042	C	T	1	ns4b	51	V	V
7126	T	A	3	ns4b	79	L	L
7177	C	T	3	ns4b	96	L	L
7225	A	G	3	ns4b	112	V	V
7246	T	C	3	ns4b	119	F	F
7258	C	T	3	ns4b	123	A	A
7279	A	G	3	ns4b	130	Q	Q
7369	C	A	3	ns4b	160	P	P
7393	T	C	3	ns4b	168	I	I
7405	A	G	3	ns4b	172	K	K
7450	A	G	3	ns4b	187	V	V
7489	T	C	3	ns4b	200	I	I
7501	T	C	3	ns4b	204	A	A
7531	A	T	3	ns4b	214	A	A
7576	T	C	3	ns4b	229	I	I
7603	G	A	3	ns4b	238	L	L
7609	G	A	3	ns4b	240	**M**	**I**
7705	T	C	3	ns5	17	N	N
7786	A	G	3	ns5	44	R	R
7792	A	G	3	ns5	46	E	E
7849	G	A	3	ns5	65	L	L
7867	T	C	3	ns5	71	L	L
7990	G	A	3	ns5	112	E	E
8056	T	C	3	ns5	134	Y	Y
8305	T	C	3	ns5	217	H	H
8335	A	C	3	ns5	227	G	G
8465	T	C	1	ns5	271	L	L
8635	C	T	3	ns5	327	V	V
8680	C	T	3	ns5	342	T	T
8752	A	T	3	ns5	366	P	P
8919	G	A	2	ns5	422	**R**	**K**
8949	G	A	2	ns5	432	**R**	**K**
8959	A	G	3	ns5	435	E	E
8971	A	G	3	ns5	439	E	E
9001	C	T	3	ns5	449	C	C
9013	T	C	3	ns5	453	I	I
9079	C	T	3	ns5	475	A	A
9082	C	T	3	ns5	476	I	I
9355	T	C	3	ns5	567	A	A
9442	C	T	3	ns5	596	I	I
9490	A	G	3	ns5	612	L	L
9661	T	C	3	ns5	669	D	D
9703	C	T	3	ns5	683	L	L
9869	C	T	1	ns5	739	L	L
9983	T	C	1	ns5	777	L	L
9991	C	T	3	ns5	779	L	L
10024	G	A	3	ns5	790	V	V
10033	C	A	3	ns5	793	V	V
10084	A	G	3	ns5	810	T	T
10177	T	C	3	ns5	841	V	V
10222	C	T	3	ns5	856	I	I
10291	T	C	3	3'UTR	-	-	-
10367	T	C	1	3'UTR	-	-	-
10424	C	T	1	3'UTR	-	-	-
10433	C	T	1	3'UTR	-	-	-
10490	G	A	1	3'UTR	-	-	-
10662	T	C	2	3'UTR	-	-	-
11000	G	A	1	3'UTR	-	-	-

- Indicates a nucleotide change that does not result in an alteration of the CDS as it occurs in an untranslated region (UTR). Bolded changes indicate amino acid (aa) changes

Appendix Table 3. Nucleotide (nt) changes between L3 WNV detected in serum and L3 WNV detected in Vp1

Position	Ln3 serum nt	Vp1 nt	Position in codon	Protein	Position in protein	Ln3 serum aa	Vp1 Serum aa
1	C	G	1	5'UTR	N/A	N/A	N/A
114	A	T	3	capsid	24	G	G
376	G	T	1	capsid	112	**A**	**S**
682	A	G	1	prM	91	**R**	**G**
804	C	T	3	prM	131	N	N
883	T	G	1	prM	158	**L**	**V**
885	A	G	3	prM	158	L	L
888	C	A	3	prM	159	L	L
889	C	T	1	prM	160	L	L
1203	G	A	3	E	97	V	V
1911	T	C	3	E	333	D	D
1986	G	C	3	E	358	V	V
2043	T	C	3	E	377	P	P
2238	A	C	3	E	442	V	V
2250	T	C	3	E	446	F	F
2317	C	T	1	E	469	**L**	**F**
2418	C	T	3	ns1	1	D	D
2667	T	C	3	ns1	84	I	I
2853	G	C	3	ns1	146	V	V
2856	C	A	3	ns1	147	**S**	**R**
2858	G	T	2	ns1	148	**R**	**I**
2872	A	C	1	ns1	153	**M**	**L**
2904	G	T	3	ns1	163	**M**	**I**
2928	A	C	3	ns1	171	V	V
3102	C	T	3	ns1	229	H	H
3154	T	C	1	ns1	247	L	L
3447	G	C	3	ns1	344	T	T
3450	G	T	3	ns1	345	**L**	**F**
3453	T	G	3	ns1	346	V	V
3474	C	T	3	ns2a	1	Y	Y
3615	C	T	3	ns2a	48	G	G
3754	C	T	1	ns2a	95	L	L
4158	G	T	3	ns2a	229	R	R
4353	G	C	3	ns2b	63	**E**	**D**
4473	C	T	3	ns2b	103	V	V
4476	C	T	3	ns2b	104	C	C
4510	T	C	1	ns2b	116	L	L
4521	T	G	3	ns2b	119	V	V
4527	G	C	3	ns2b	121	G	G
4528	T	C	1	ns2b	122	**F**	**L**
4665	C	G	3	ns3	36	A	A
4704	A	C	3	ns3	49	L	L
4814	G	C	2	ns3	86	**G**	**A**
4917	T	G	3	ns3	120	**D**	**E**
5103	C	G	3	ns3	182	**D**	**E**
5379	C	T	3	ns3	274	H	H
5472	A	C	3	ns3	305	V	V
5557	T	A	1	ns3	334	**S**	**T**
5613	C	T	3	ns3	352	I	I
5736	T	G	3	ns3	393	T	T
5943	T	A	3	ns3	462	I	I
6036	G	T	3	ns3	493	R	R
6039	C	A	3	ns3	494	I	I
6045	T	C	3	ns3	496	L	L
6548	A	G	2	ns4a	45	**E**	**G**
6651	G	A	3	ns4a	79	K	K
6696	T	C	3	ns4a	94	C	C
6902	G	T	2	ns4b	14	**S**	**I**
7341	C	A	3	ns4b	160	P	P
7383	C	A	3	ns4b	174	G	G
7413	G	T	3	ns4b	184	A	A
7449	T	G	3	ns4b	196	R	R
7461	A	C	3	ns4b	200	I	I
7503	A	T	3	ns4b	214	A	A
7512	C	T	3	ns4b	217	V	V
7548	T	C	3	ns4b	229	V	V
7575	G	A	3	ns4b	238	L	L

Position	Ln3 serum nt	Vp1 nt	Position in codon	Protein	Position in protein	Ln3 serum aa	Vp1 Serum aa
7581	C	A	3	ns4b	240	I	I
7758	A	G	3	ns5	44	R	R
7764	A	G	3	ns5	46	E	E
7962	G	A	3	ns5	112	E	E
8046	T	C	3	ns5	140	S	S
8607	G	T	3	ns5	327	V	V
8652	C	T	3	ns5	342	T	T
8724	A	T	3	ns5	366	P	P
8757	T	C	3	ns5	377	N	N
8973	C	T	3	ns5	449	C	C
9037	A	C	1	ns5	471	R	R
9038	G	C	2	ns5	471	**R**	**T**
9041	G	C	2	ns5	472	**G**	**A**
9066	G	C	3	ns5	480	**W**	**C**
9073	G	T	1	ns5	483	**A**	**S**
9327	G	C	3	ns5	567	A	A
9414	A	T	3	ns5	596	I	I
9462	A	G	3	ns5	612	L	L
9633	T	C	3	ns5	669	D	D
9996	G	A	3	ns5	790	V	V
10005	C	A	3	ns5	793	V	V
10056	C	G	3	ns5	810	T	T
10149	T	C	3	ns5	841	I	I
10567	A	T	1	3'UTR	N/A	N/A	N/A
10575	T	C	3	3'UTR	N/A	N/A	N/A
10704	C	G	3	3'UTR	N/A	N/A	N/A

- Indicates a nucleotide change that does not result in an alteration of the CDS as it occurs in an untranslated region (UTR). Bolded changes indicate amino acid (aa) changes.

Appendix Table 4. Nucleotide (nt) changes between L1 WNV detected in serum and L1 WNV detected in Vp1 and NY99

Position	NY99 nt	Serum ln1 nt	Vp1 ln1 nt	Position in codon	Protein	Position in protein	NY99 aa	Serum ln1 aa	Vp1 ln1 aa
55	C	T	T	1	5'UTR	5'UTR	-	-	-
62	C	T	T	2	5'UTR	5'UTR	-	-	-
89	A	G	G	2	5'UTR	5'UTR	-	-	-
288	A	G	G	3	capsid	64	A	A	A
340	C	T	T	1	capsid	82	L	L	L
435	C	T	C	3	capsid	113	V	V	V
525	A	T	A	3	prM	20	T	T	T
642	T	C	T	3	prM	59	G	G	G
858	C	C	T	3	prM	131	N	N	N
1257	G	G	A	3	E	97	V	V	V
1308	C	T	C	3	E	114	D	D	D
1368	T	T	C	3	E	134	N	N	N
1395	C	T	C	3	E	143	V	V	V
1401	A	A	G	3	E	145	G	G	G
1442	T	C	C	2	E	159	**V**	**A**	**A**
1473	C	T	C	3	E	169	I	I	I
1494	C	C	T	3	E	176	Y	Y	Y
1662	G	A	G	3	E	232	V	V	V
1731	G	G	A	3	E	255	L	L	L
1750	C	T	C	1	E	262	L	L	L
1794	C	C	T	3	E	276	S	S	S
1893	C	C	T	3	E	309	F	F	F
1939	T	T	C	1	E	325	L	L	L
1965	T	T	C	3	E	333	D	D	D
2040	C	T	C	3	E	358	V	V	V
2097	A	A	C	3	E	377	P	P	P
2109	C	T	C	3	E	381	D	D	D
2292	C	T	C	3	E	442	V	V	V
2304	C	T	C	3	E	446	F	F	F
2371	C	C	T	1	E	469	L	L	L
2466	C	T	T	3	E	500	H	H	H
2472	C	C	T	3	ns1	1	D	D	D
2502	A	G	A	3	ns1	11	Q	Q	Q

Position	NY99 nt	Serum ln1 nt	Vp1 ln1 nt	Position in codon	Protein	Position in protein	NY99 aa	Serum ln1 aa	Vp1 ln1 aa
2586	A	A	G	3	ns1	39	P	P	P
2593	C	C	T	1	ns1	42	L	L	L
2607	T	C	T	3	ns1	46	I	I	I
2751	G	A	G	3	ns1	94	E	E	E
2926	T	T	C	1	ns1	153	L	L	L
2958	C	C	T	3	ns1	163	T	T	T
2982	C	T	C	3	ns1	171	V	V	V
3138	T	T	C	3	ns1	223	C	C	C
3156	T	C	T	3	ns1	229	H	H	H
3204	C	C	T	3	ns1	245	V	V	V
3208	C	T	C	1	ns1	247	L	L	L
3444	T	C	T	3	ns1	325	T	T	T
3501	C	T	C	3	ns1	344	T	T	T
3504	C	C	T	3	ns1	345	L	L	L
3507	G	A	G	3	ns1	346	V	V	V
3528	T	C	T	3	ns2a	1	Y	Y	Y
3669	C	C	T	3	ns2a	48	G	G	G
3680	C	C	T	2	ns2a	52	**T**	**T**	**I**
3693	C	T	C	3	ns2a	56	R	R	R
3699	C	C	T	3	ns2a	58	V	V	V
3703	T	T	C	1	ns2a	60	L	L	L
3729	T	C	T	3	ns2a	68	S	S	S
3808	C	C	T	1	ns2a	95	**L**	**L**	**F**
3880	C	C	T	1	ns2a	119	**H**	**H**	**Y**
3912	C	T	C	3	ns2a	129	I	I	I
3960	C	C	T	3	ns2a	145	A	A	A
3969	C	T	T	3	ns2a	148	F	F	F
4107	C	T	T	3	ns2a	194	A	A	A
4110	A	G	A	3	ns2a	195	A	A	A
4146	A	G	G	3	ns2a	207	L	L	L
4212	T	C	T	3	ns2a	229	R	R	R
4527	C	C	T	3	ns2b	103	V	V	V
4530	T	C	T	3	ns2b	104	C	C	C
4564	T	T	C	1	ns2b	116	L	L	L
4611	A	G	G	3	ns2b	131	R	R	R
4680	C	C	T	3	ns3	23	Y	Y	Y
4719	A	A	G	3	ns3	36	A	A	A
4725	G	G	A	3	ns3	38	A	A	A
4749	C	T	C	3	ns3	46	F	F	F
4758	T	T	C	3	ns3	49	L	L	L
4803	C	T	T	3	ns3	64	R	R	R
4830	G	G	A	3	ns3	73	K	K	K
4848	C	C	T	3	ns3	79	Y	Y	Y
4944	G	G	A	3	ns3	111	T	T	T
4955	T	C	T	2	ns3	115	**V**	**A**	**V**
4962	A	A	G	3	ns3	117	K	K	K
4971	A	A	G	3	ns3	120	E	E	E
4986	C	T	T	3	ns3	125	A	A	A
5082	C	T	C	3	ns3	157	P	P	P
5148	C	C	T	3	ns3	179	F	F	F
5157	G	T	G	3	ns3	182	**E**	**D**	**E**
5280	G	A	A	3	ns3	223	A	A	A
5400	T	C	T	3	ns3	263	H	H	H
5430	T	T	C	3	ns3	273	P	P	P
5433	C	C	T	3	ns3	274	H	H	H
5457	C	T	C	3	ns3	282	F	F	F
5526	C	T	C	3	ns3	305	V	V	V
5611	T	T	A	1	ns3	334	**S**	**S**	**T**
5658	C	C	T	3	ns3	349	Y	Y	Y
5667	C	C	T	3	ns3	352	I	I	I
5678	C	C	T	2	ns3	356	**T**	**T**	**I**
5790	G	T	G	3	ns3	393	T	T	T
5958	A	G	G	3	ns3	449	A	A	A
5964	A	G	G	3	ns3	451	T	T	T
5973	T	C	C	3	ns3	454	S	S	S
5994	T	C	C	3	ns3	461	R	R	R
5997	C	T	T	3	ns3	462	I	I	I
6030	C	T	T	3	ns3	473	Y	Y	Y

Position	NY99 nt	Serum ln1 nt	Vp1 ln1 nt	Position in codon	Protein	Position in protein	NY99 aa	Serum ln1 aa	Vp1 ln1 aa
6102	C	T	T	3	ns3	497	D	D	D
6138	C	T	T	3	ns3	509	F	F	F
6204	A	G	G	3	ns3	531	R	R	R
6258	C	T	T	3	ns3	549	Y	Y	Y
6375	A	G	A	3	ns3	588	E	E	E
6426	C	T	T	3	ns3	605	H	H	H
6468	T	T	C	3	ns3	619	R	R	R
6522	G	G	A	3	ns4a	18	G	G	G
6540	T	C	T	3	ns4a	24	L	L	L
6543	C	C	T	3	ns4a	25	D	D	D
6588	C	C	T	3	ns4a	40	H	H	H
6598	C	C	T	1	ns4a	44	L	L	L
6618	T	C	T	3	ns4a	50	A	A	A
6705	G	G	A	3	ns4a	79	K	K	K
6712	T	T	C	1	ns4a	82	L	L	L
6750	T	T	C	3	ns4a	94	C	C	C
6895	C	C	T	1	ns4a	143	**L**	**L**	**F**
6901	A	A	G	1	ns4a	145	**S**	**S**	**G**
6956	G	G	T	2	ns4b	14	**S**	**S**	**I**
6996	C	T	T	3	ns4b	27	F	F	F
7068	C	C	T	3	ns4b	51	V	V	V
7152	A	T	A	3	ns4b	79	L	L	L
7203	C	C	T	3	ns4b	96	L	L	L
7251	A	A	G	3	ns4b	112	V	V	V
7272	T	T	C	3	ns4b	119	F	F	F
7284	C	C	T	3	ns4b	123	A	A	A
7305	A	A	G	3	ns4b	130	Q	Q	Q
7395	A	C	A	3	ns4b	160	P	P	P
7419	C	T	C	3	ns4b	168	I	I	I
7431	A	A	G	3	ns4b	172	K	K	K
7476	A	A	G	3	ns4b	187	V	V	V
7515	T	T	C	3	ns4b	200	I	I	I
7527	C	T	C	3	ns4b	204	A	A	A
7557	A	A	T	3	ns4b	214	A	A	A
7602	C	T	C	3	ns4b	229	I	I	I
7629	A	G	A	3	ns4b	238	L	L	L
7635	A	G	A	3	ns4b	240	**I**	**M**	**I**
7731	C	T	C	3	ns5	17	N	N	N
7812	G	A	G	3	ns5	44	R	R	R
7818	A	A	G	3	ns5	46	E	E	E
7866	G	G	A	3	ns5	62	L	L	L
7893	C	T	C	3	ns5	71	L	L	L
7938	T	C	C	3	ns5	86	G	G	G
8016	G	G	A	3	ns5	112	E	E	E
8082	C	T	C	3	ns5	134	Y	Y	Y
8235	T	C	C	3	ns5	185	C	C	C
8331	C	T	C	3	ns5	217	H	H	H
8361	C	A	C	3	ns5	227	G	G	G
8491	C	T	C	1	ns5	271	L	L	L
8511	T	A	A	3	ns5	277	**S**	**R**	**R**
8661	C	C	T	3	ns5	327	V	V	V
8706	C	C	T	3	ns5	342	T	T	T
8778	T	A	T	3	ns5	366	P	P	P
8945	G	G	A	2	ns5	422	**R**	**R**	**K**
8975	A	G	A	2	ns5	432	**K**	**R**	**K**
8985	G	A	G	3	ns5	435	E	E	E
8997	G	A	G	3	ns5	439	E	E	E
9027	T	C	T	3	ns5	449	C	C	C
9039	T	T	C	3	ns5	453	I	I	I
9105	C	C	T	3	ns5	475	A	A	A
9108	T	C	T	3	ns5	476	I	I	I
9352	C	T	T	1	ns5	558	L	L	L
9381	C	T	C	3	ns5	567	A	A	A
9468	C	C	T	3	ns5	596	I	I	I
9516	A	A	G	3	ns5	612	L	L	L
9687	C	T	C	3	ns5	669	D	D	D
9729	C	C	T	3	ns5	683	L	L	L
9895	T	C	T	1	ns5	739	L	L	L

Position	NY99 nt	Serum ln1 nt	Vp1 ln1 nt	Position in codon	Protein	Position in protein	NY99 aa	Serum ln1 aa	Vp1 ln1 aa
10009	C	T	C	1	ns5	777	L	L	L
10017	C	C	T	3	ns5	779	L	L	L
10050	G	G	A	3	ns5	790	V	V	V
10059	C	C	A	3	ns5	793	V	V	V
10110	A	A	G	3	ns5	810	T	T	T
10203	C	T	C	3	ns5	841	V	V	V
10248	T	C	T	3	ns5	856	I	I	I
10377	C	T	C	3	3'UTR	-	-	-	-
10398	C	T	T	3	3'UTR	-	-	-	-
10453	C	T	C	1	3'UTR	-	-	-	-
10468	C	T	T	1	3'UTR	-	-	-	-
10495	C	T	T	1	3'UTR	-	-	-	-
10510	T	C	T	1	3'UTR	-	-	-	-
10519	T	C	T	1	3'UTR	-	-	-	-
10576	G	A	G	1	3'UTR	-	-	-	-
10749	T	C	T	3	3'UTR	-	-	-	-

Genbank accession NY99 - MZ605381. - Indicates a nucleotide change that does not result in an alteration of the CDS as it occurs in an untranslated region (UTR). Bolded changes indicate amino acid (aa) changes

Appendix Table 5. Nucleotide (nt) changes between L3 WNV detected in serum and L3 WNVs submitted to Genbank

Position	22a serum nt	97-103 nt	99-222 nt	06-222 nt	Position in codon	Protein	Position in protein	22a serum aa	97-103 aa	99-222 aa	06-222 aa
30	T	C	C	T	3	5'UTR	-	-	-	-	-
528	A	A	A	G	3	prM	39	**I**	**I**	**I**	**M**
555	C	C	T	C	3	prM	48	**T**	**T**	**T**	**T**
666	A	C	A	A	3	prM	85	R	R	R	R
831	C	C	T	C	3	prM	140	V	V	V	V
1276	A	A	G	A	1	E	122	**T**	**T**	**A**	**T**
1545	T	T	C	T	3	E	211	F	F	F	F
1962	T	T	C	T	3	E	350	T	T	T	T
2094	C	C	T	C	3	E	394	N	N	N	N
2180	C	A	C	C	2	E	423	**A**	**D**	**A**	**A**
2559	G	G	A	G	3	ns1	48	K	K	K	K
2895	C	C	T	C	2	ns1	160	F	F	F	F
5662	G	A	No cov	No cov	1	ns3	369	**G**	**S**	No cov	No cov
5885	T	C	No cov	No cov	2	ns3	443	**I**	**T**	No cov	No cov
6847	G	A	No cov	No cov	1	ns4a	145	**G**	**S**	No cov	No cov
7786	C	T	No cov	No cov	1	ns5	54	**P**	**S**	No cov	No cov
9082	T	C	No cov	No cov	1	ns5	486	L	L	No cov	No cov
9576	G	C	No cov	No cov	3	ns5	650	T	T	No cov	No cov
9789	C	G	No cov	No cov	3	ns5	721	L	L	No cov	No cov
10633	G	G	No cov	No cov	1	3'UTR	-	-	-	No cov	No cov
10691	C	T	No cov	No cov	2	3'UTR	-	-	-	No cov	No cov

Genbank accession numbers: 97-103 (AY765264), 99-222 (GQ421358) and 06-222(GQ421359). - Indicates a nucleotide change that does not result in an alteration of the CDS as it occurs in an untranslated region (UTR). Bolded changes indicate amino acid (aa) changes. No cov indicates there was no reported nt at the indicated position in the Genbank reference

Appendix Table 6. Nucleotide (nt) changes between L3 WNV detected in serum and L3 WNV detected in CSF

Position	Ln3 serum nt	Ln3 CSF nt	Position in codon	Protein	Position in protein	Ln3 serum aa	Ln3 CSF aa
3129	G	T	3	ns1	238	**E**	**D**
3295	C	A	1	ns1	294	**R**	**S**
3896	T	G	2	ns2a	142	**V**	**G**
3932	T	G	2	ns2a	154	**V**	**G**
4049	C	A	2	ns2a	193	**A**	**E**
6281	A	G	2	ns3	575	**E**	**G**
6336	G	C	3	ns3	593	**R**	**S**
6341	G	C	2	ns3	595	**R**	**P**
6346	G	C	1	ns3	597	**A**	**P**

Bolded changes indicate amino acid (aa) changes.

Appendix Table 7. Plaque pick composition

Plaque pick	Temperature grown (ºC)	Days post overlay	Lineage 1 WNV (% genome coverage)	Lineage 3 WNV (% genome coverage)
1	32	3	100	56.6
2	32	9	100	83.5
3	28	7	100	90.4

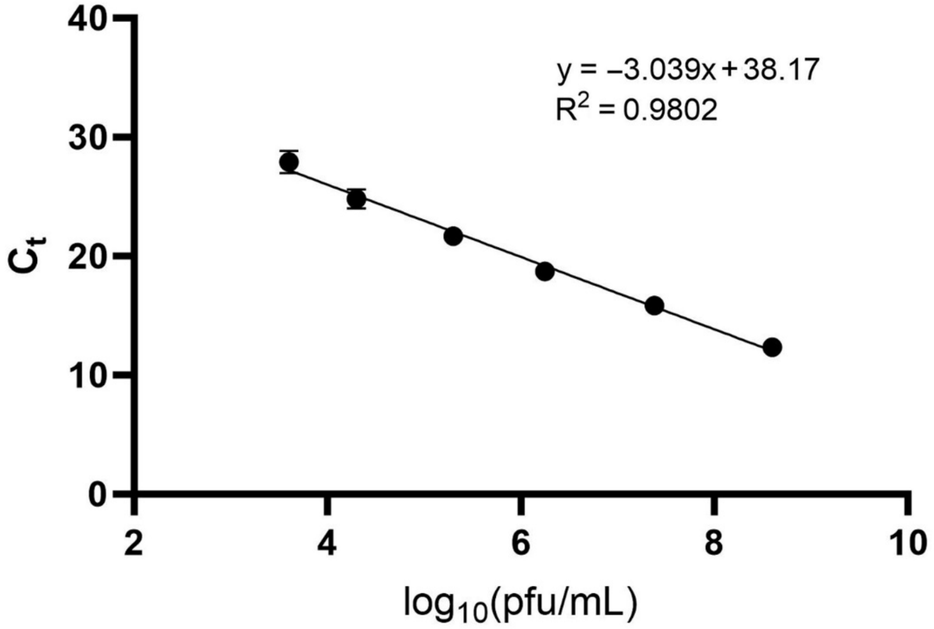

Appendix Figure. NY99 standard curve. Lineage 1 WNV strain, NY99, was serially diluted and quantified using plaque assay and rRT-PCR after RNA extraction. The standard curve was quantified using a best-fit line model.
